# Chest wall motion analysis in healthy volunteers and adults with cystic fibrosis using a novel Kinect-based motion tracking system

**DOI:** 10.1007/s11517-015-1433-1

**Published:** 2016-02-13

**Authors:** James M. Harte, Christopher K. Golby, Johanna Acosta, Edward F. Nash, Ercihan Kiraci, Mark A. Williams, Theodoros N. Arvanitis, Babu Naidu

**Affiliations:** 1 Institute of Digital Healthcare, WMG, University of Warwick, Coventry, UK; 2 Interacoustics Research Unit, c/o Technical University of Denmark, Bldg. 351, Kongens Lyngby, 2800 Denmark; 3 Heart of England NHS Foundation Trust, Birmingham, UK; 4 WMG, University of Warwick, Coventry, UK; 5 University of Birmingham, Birmingham, UK

**Keywords:** Thoracic wall, Chest wall, Thoracic surgery, Respiratory system diagnostic technique, Medical device design

## Abstract

Respiratory disease is the leading cause of death in the UK. Methods for assessing pulmonary function and chest wall movement are essential for accurate diagnosis, as well as monitoring response to treatment, operative procedures and rehabilitation. Despite this, there is a lack of low-cost devices for rapid assessment. Spirometry is used to measure air flow expired, but cannot infer or directly measure full chest wall motion. This paper presents the development of a low-cost chest wall motion assessment system. The prototype was developed using four Microsoft Kinect sensors to create a 3D time-varying representation of a patient’s torso. An evaluation of the system in two phases is also presented. Initially, static volume of a resuscitation mannequin with that of a Nikon laser scanner is performed. This showed the system has slight underprediction of 0.441 %. Next, a dynamic analysis through the comparison of results from the prototype and a spirometer in nine cystic fibrosis patients and thirteen healthy subjects was performed. This showed an agreement with correlation coefficients above 0.8656 in all participants. The system shows promise as a method for assessing respiratory disease in a cost-effective and timely manner. Further work must now be performed to develop the prototype and provide further evaluations.

## Introduction

Respiratory disease, including lung cancer, is the leading cause of death in the UK, accounting for 920,000 disability-adjusted life years lost [10]. It is the most frequent cause of disease in primary care in all age groups and the second most common cause of chronic conditions.

For patients who report to A&E, a quick and low-cost method of assessment is required. One process currently used to assess respiratory disease in a low-cost and time-effective manner is spirometry [14]. Spirometry allows for the measurement of expired airflow from the lungs, enabling physicians to better characterise the cause of breathlessness and to assess progression of respiratory disease over time.

However, spirometry can have significant limitations. Firstly, forced spirometric efforts allow assessment of initial diaphragm/muscle strength and can only measure total airflow in and out of the lungs; it therefore provides a limited amount of feedback and does not allow physicians to identify motion at the chest and the relative contribution of different areas of each lung to the subjects’ respiratory function [16]. This is particularly important in subjects with more focal lung abnormalities, such as emphysematous bullae, collapsed lung segments and previous surgical lung resection. Secondly, the effect of chest wall abnormalities, such as respiratory muscle weakness and pectus excavatum, as well as diaphragm movement cannot be assessed by spirometry [21]. Thirdly, since it is an effort-dependent procedure, there is a potential for inaccurate results in subjects unable to reliably perform a forced expiratory manoeuvre [12] (e.g. children, the elderly and subjects with hearing impairments, learning difficulties or a language barrier). Fourthly, subjects with facial abnormalities or muscle weakness are often unable to form a tight seal around the mouthpiece, preventing spirometry being accurately performed [8].

As a result of these limitations, there has been increasing interest in the development of alternative methods of assessing respiratory function, including chest wall motion analysis. Systems such as magnetometers [17], respiratory inductance plethysmography [13], optoelectronic plethysmography [1, 4] (OEP) and structured light plethysmography [5] (SLP) have been demonstrated for this purpose; however, these technologies have been shown can be cumbersome, expensive, time consuming, difficult to interpret and not suited to the clinical environment. A more recent method for evaluating chest wall motion is through the use of the Microsoft Kinect technology (a low-cost motion tracking camera). This system is portable, low cost, non-invasive and has been suggested as an alternative for spirometry [3] with positive correlations being shown (Ye et al. 2012 show correlations of *r* = 0.966 [24]). However, research demonstrating the use of this technology utilises a one-camera version of the system [3, 7, 23, 24]. This method follows a process of monitoring the chest wall only and detecting changes in surface. The issue with this is that motion may affect the sample, particularly large movements during analysis. In addition, it is difficult to compartmentalise different parts of the torso and also calculate volumes of the whole or parts of the torso.

This paper reports the development of a low-cost and time-effective novel prototype for capturing dynamic chest wall motion using four Microsoft Kinect sensors. An initial evaluation of this prototype is also presented, involving healthy volunteers and adults with cystic fibrosis (an inherited condition causing progressive respiratory failure) [20].

## Methods

### System design

This research created a system which was capable of assessing respiratory motion using a low-cost and time-effective motion tracking device. The device to be used was the Microsoft Kinect, which is a human tracking peripheral used in the gaming industry for the Microsoft Xbox to provide low-cost 3D motion capture capabilities [6]. The device is composed of an infrared (IR) laser projector, an IR camera, a colour camera and a microphone array. The Kinect uses the IR projector to detect distance of an object from the sensor by emitting a single infrared beam which is split to create an invisible pattern of speckles [11]. This pattern is captured by the IR camera and compared against a reference pattern stored in the device to calculate distance from external objects. This study utilised four of these devices to create three-dimensional representations of a subject’s torso over time, so difference could be calculated by comparing the difference between frames.

### System evaluation: static analysis methods

In order to evaluate the derived system, static testing was initially performed to evaluate overall accuracy of the system. A cardiopulmonary resuscitation (CPR) training mannequin was used as the evaluation object, of which volume could be calculated. This was a torso-type mannequin measuring 63.5 cm in height and weighing 3.9 kg.

Initially, the mannequin was recorded five times by the developed, Kinect-based system in a room with mainly artificial light. Each capture lasted approximately 15 s.

Next, a Nikon Metrology MCA 2400 M7 articulated arm laser scanner (Nikon, Tokyo, Japan) was used to capture complete detailed geometric scans. Single point accuracy of the scanner is 0.031 mm, and length accuracy is 0.042 mm with an uncertainty of ±*2σ*. A typical scan path for the laser device can capture up to 72,000 points per second.

Results from the two scans were then compared for static volume estimation.

To make a comparison between the Kinect-based system and Nikon laser scanner, initially a root mean square (RMS) error was calculated, including mean and mode. Probability density and cumulative density function are then also utilised to assess estimate errors and bias.

### System evaluation: dynamic capture methods

Chest wall motion was measured with the developed, Kinect-based system, whilst participants simultaneously performed spirometry using a MicroLab Spirometer (CareFusion Corp, San Diego, USA).

Spirometry was performed according to ATS/ERS guidelines [14]. CareFusion Spirometry PC Software version 1.07 (CareFusion Corp, San Diego, USA) was used to store patient information and spirometry results.

Nine subjects with cystic fibrosis were recruited at the West Midlands Adult Cystic Fibrosis Centre and 13 healthy volunteers from staff at Heart of England NHS Foundation Trust.

The acquisition protocol involved capturing quiet breathing for 20 s, followed by a relaxed vital capacity (VC) manoeuver (maximum inspiration and expiration) and followed by 20 s of quiet breathing. The test was run three times per patient, as per ATS/ERS guidelines. Males were captured bare-chested, whilst females wore tight t-shirts for modesty. From total chest wall volumes acquired by the Kinect-based device, the following parameters were obtained: tidal volume (VT), as the total chest wall volume variation, respiratory rate (RR) and minute ventilation (VT × RR).

In order to process the spirometry readings for analysis, some data required additional processing. Initially, spirometry data and time stamps were imported into MATLAB. The time series captured needed a shape-preserving piecewise cubic interpolation procedure to ensure a constant sampling rate (5 Hz). A fourth-order zero-phase band-pass Butterworth filter [0.025–1 Hz] was applied.

The two volume time series were aligned by searching for the peak in their cross-correlation function. A measure of similarity was needed to compare the two time series over a window of interest. The range for comparison was set at −8 to 14 s relative to the maximum point on the relaxed VC measurement. This window length was the largest possible to ensure all subjects tested had sufficient overlap in the time series recorded. The Pearson product moment correlation coefficient was chosen as a simple measure of similarity:

$$\rho = \frac{{\text{cov} \left( {v_{\text{K}} ,v_{\text{S}} } \right)}}{{\sigma_{\text{K}} \sigma_{\text{S}} }}$$

where $\text{cov} (v_{\text{K}} ,v_{\text{S}} )$ is covariance between the Kinect-based and spirometry-based volume estimates and the $\sigma_{\text{K}}$ and *σ*_S_ are their respective standard deviations. It takes values at ±1 and at its extremes this corresponds to data points lying perfectly on a line. A summary of all of the correlation coefficients obtained between the Kinect-based and spirometry measurements for the two groups (healthy subjects and CF patients) is presented. This is followed by a one-way ANOVA to compare sample means between the two groups. Finally, a TLS fitting procedure is used to compare results from the Kinect-based system and spirometry measurements.

The research study was approved by the Local Research Ethics Committee (*reference number: 10/H1202/58*). Informed consent was obtained from all involved participants, and the study conforms to the Declaration of Helsinki.

## Results

### System implementation

**Fig. 1:**
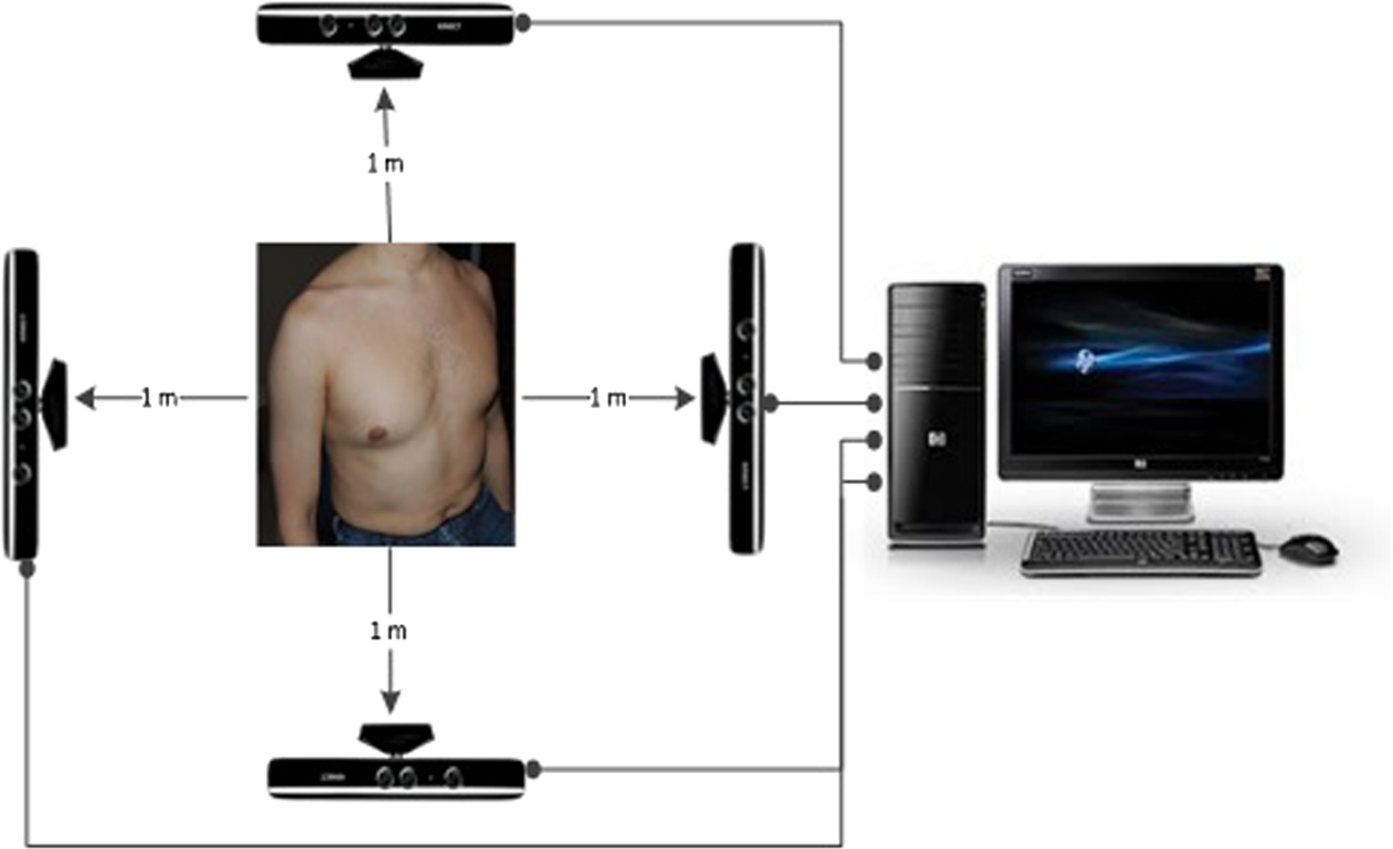
System overview

To enable chest wall motion analysis, a system comprising four Kinect sensors was developed and orientated as shown in Fig. 1. The Kinect sensors were placed around the subject, ensuring a minimum distance of 1 metre, following recommendations from Alnowami et al. [2].

Each Kinect sensor was connected to a PC using an independent and dedicated USB bus. The general settings of the system allowed for 30 frames per second to be captured in 320 × 240 pixels for each sensor.

A bespoke software application was designed, using the Kinect Software Development Kit (SDK) 1.6 (Microsoft, Redmond, USA) which assisted the process.

In order to calibrate the system, a static object was placed in the middle of the four Kinects. The bespoke software was used to take a scan using each Kinect individually, with the other cameras turned off, to avoid interference from competing infrared laser projectors. The four separate scans taken from each Kinect were then exported from the bespoke software and imported into the meteorology software Geomagic Studio 2012. This software allowed for the four images to be aligned using in-built functionality to map the four images, and form a point cloud. This resulted in a transformation matrix being created which detailed the transformations of each of the original four scans. These were then exported back to the bespoke software.

At this point, a patient could be placed in between the four cameras and a recording taken of their torso, using the bespoke software. The images at each frame, from each camera, were combined using the previously generated transformation matrix from the calibration step. This formed a set of 3D point clouds at each frame captured, from which change in volume could be calculated, by comparing the point cloud at each frame.

**Fig. 2:**
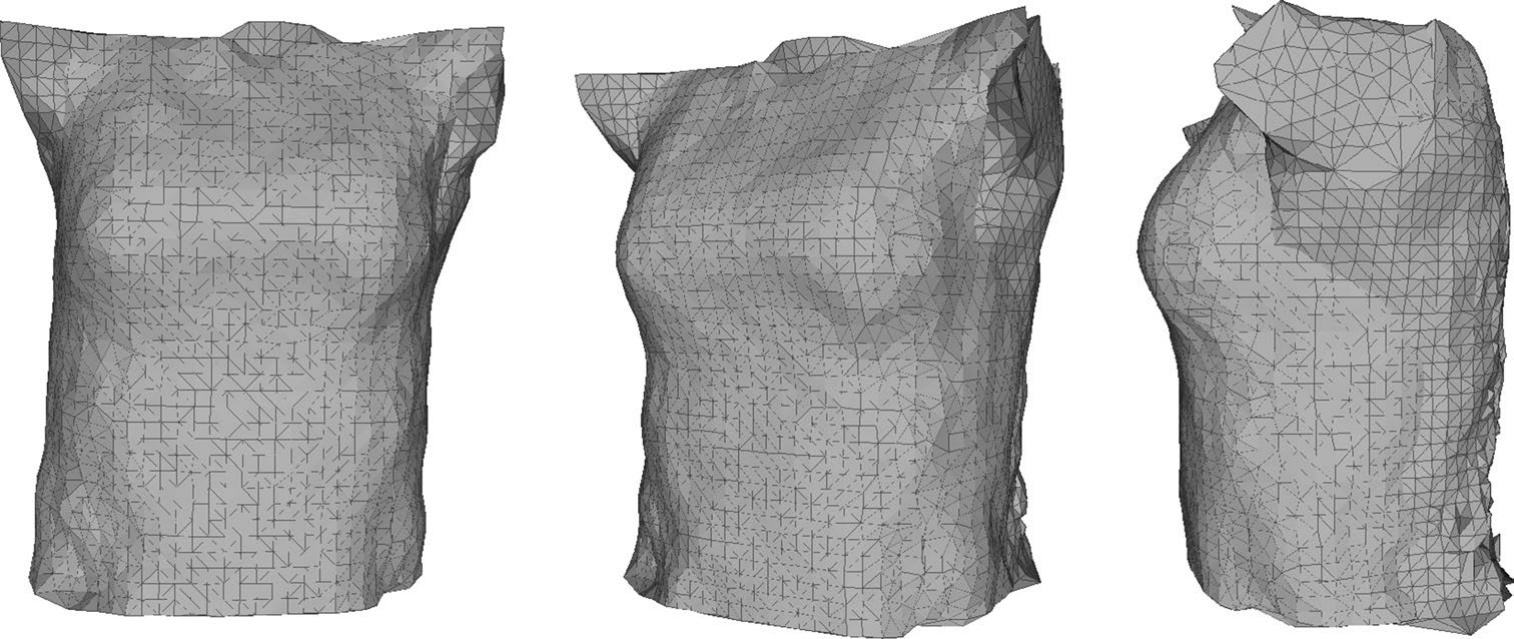
Scan taken by the Kinect-based system

In order to perform this calculation, Geomagic Studio 2012 was utilised again, using a set macro which was created. This allowed for a repetition of the same task, which included loading each of the point clouds which were exported from the bespoke software, building a 3D mesh (triangles and normals), filling any holes in the mesh and using the mesh doctor to smooth the resulting object. The user is asked to manually define the limits of the analysis before this macro is run, placing a bounding box around an initial image to limit volume calculations to the torso only. These limits are then applied to all meshes within the analysis. This process also helps to eliminate noise from outside the bounding box. An example of a scan taken of a mannequin is shown in Fig. 2.

**Fig. 3:**
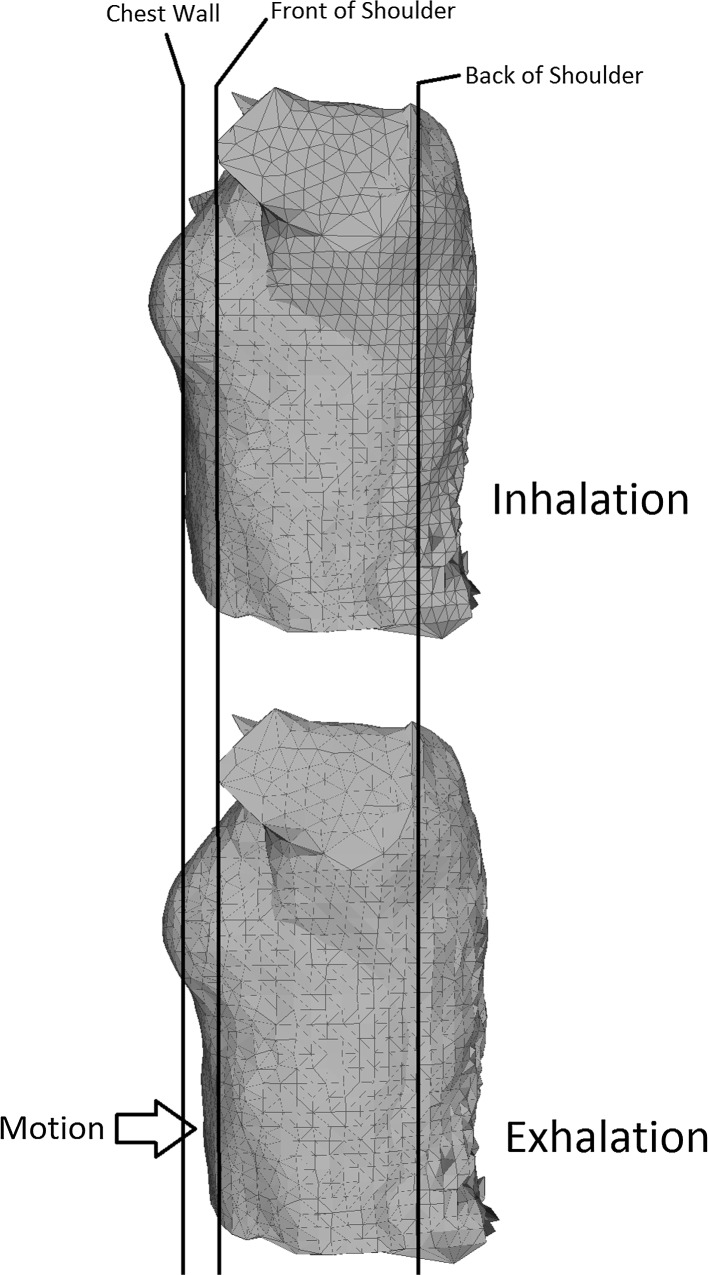
Change in volume as captured by the Kinect-based system

A 3D model is created for each subsequent frame, and this allows change in volume in the torso to be detected. An example of the change in volume in a model is shown in Fig. 3.

In order to achieve this, a ply file was stored for each point cloud. 3D volume data and the individual frame time stamps for all four Kinect sensors were exported from Geomagic and imported into MATLAB R2012B (version 8.0) (MathWorks, Natick, USA). Volumes were calculated using a fast algorithm in MATLAB [19]. Occasional misalignment with the four camera time stamps caused errors in the 3D reconstruction of the chest wall volume. This occurred as the clocks for the four Kinect sensors cannot be synchronised with the drivers supplied in the Microsoft SDK. An algorithm was implemented to detect sudden sharp changes in volume, making the assumption that these were artefacts. The resulting 3D volume time series did not have a constant sampling rate, due to both the artefact removal process and the way the Kinect sensor captures data. Therefore, a shape-preserving, piecewise cubic interpolation procedure [9] was used to construct from the original time series one with a constant sampling rate of 30 Hz. A fourth-order zero-phase band-pass Butterworth filter [22] [0.025–1 Hz] was used to remove noise and emphasise the frequency region of interest, and a further down-sampling procedure was applied to reduce the sampling rate to 5 Hz. This also removed the static volume of each subject under test and only estimated the dynamic volume.

### System evaluation: static analysis results

The mannequin was successfully scanned by the Nikon Metrology laser scanner and the Kinect-based system.

The estimates from the Kinect-based system were processed, and the gold standard reference volume (22.751 l) from the Nikon Metrology scanner was subtracted. The root mean square (RMS) error for the Kinect-based estimate was $\varepsilon_{\text{RMS}} = 0.100$ litres to three decimal places, corresponding to an error of 0.441 %. The mean of the error is 0.0626 l and the mode (most common value) 0.0216 l.

**Fig. 4:**
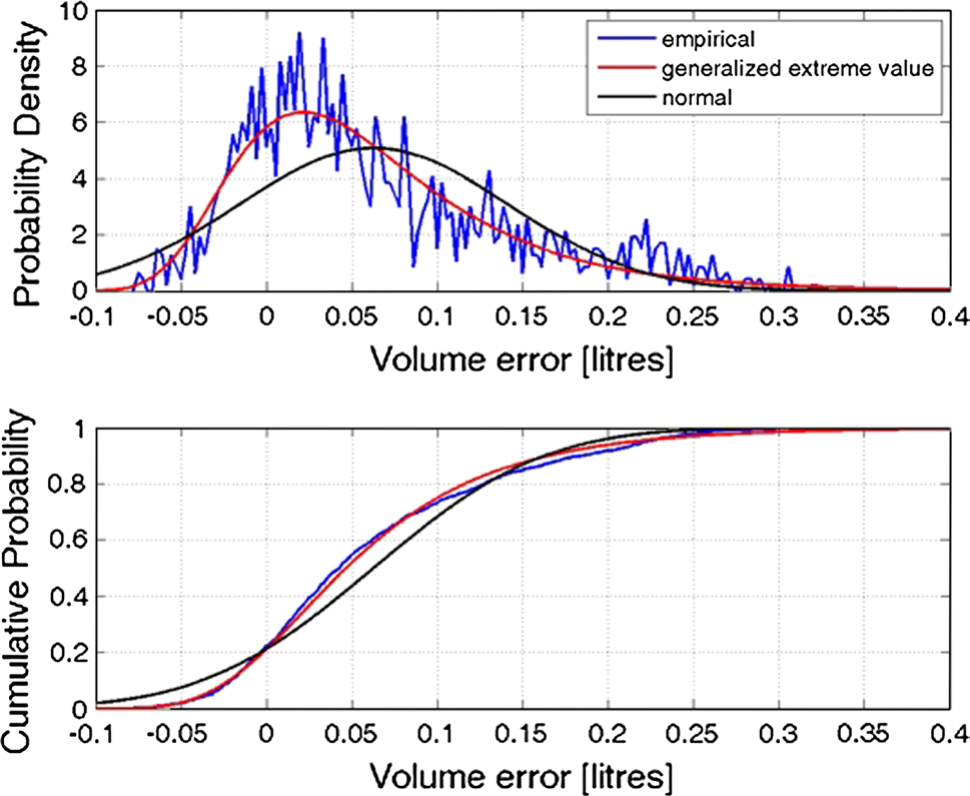
Probability density function and cumulative density function for mannequin data

Figure 4 shows the empirical probability density and cumulative density function (CDF) (blue curves) of the Kinect-based volume estimate errors. It can be seen that the error distribution is skewed with a long right tail (indicating underestimation) and has a nonzero mean and mode (indicating bias). Taken together, these make it more likely that the volume is underestimated with the Kinect sensors. A series of probability distributions were fitted, and the best was chosen based on Akaike information criterion (AIC). This corresponded to a generalised extreme value distribution, as it could adequately model the long right tail seen in the empirical results. Figure 4 also shows the fitted generalised extreme value distribution (red curve) and a fitted normal/Gaussian distribution (black curve) for reference.

The probability density function for the generalised extreme value distribution was given by:

$$\begin{aligned} f\left( {\varepsilon |\mu ,\sigma ,k} \right) & = \left( {\frac{1}{\sigma }} \right)\exp \left( { - \left( {1 + k\frac{{\left( {\varepsilon - \mu } \right)}}{\sigma }} \right)^{{ - \frac{1}{k}}} } \right) \\ & \quad \times \left( {1 + k\frac{{\left( {\varepsilon - \mu } \right)}}{\sigma }} \right)^{{ - \left( {k + 1} \right)/k}} \\ \end{aligned}$$

where $\mu \in$ is a location parameter, $\sigma > 0$ a scale parameter and $k \in$ a shape parameter. The fitted parameter values and their lower and upper 95 % confidence intervals (CI) were found to be:

- *μ* = 0.0252 [0.0220, 0.0284] (location);
- *σ* = 0.0580 [0.0557, 0.060] (scale);
- *k* = 0.0641 [0.0231, 0.1051] (shape).

The cumulative density function was given by:

$$F\left( {\varepsilon |\mu ,\sigma ,k} \right) = \exp \left( { - \left( {1 + k\frac{{\left( {\varepsilon - \mu } \right)}}{\sigma }} \right)^{ - 1/k} } \right)$$

In order to derive error bounds for the distribution, the CDF was inverted to give $\varepsilon < \mu + \frac{\sigma }{k}\left( {\left( {\ln \frac{1}{F}} \right)^{ - k} - 1} \right)$ (i.e. the volume error for a given cumulative probability). Thus, the error $\varepsilon < 0.215$, with a probability of (*F*=) 0.95, and the error $\varepsilon < - 0.0363$, with a probability of 0.05, were obtained. It is thus possible to define the 95 % error bounds for static volume estimation as $- 0.0363 < \varepsilon < 0.2150$.

### System evaluation: dynamic capture results

Scans using the developed system, and simultaneous spirometry readings, were successfully taken of nine subjects with cystic fibrosis and 13 healthy volunteers. Table 1 presents the demographics of the study participants.

**Table 1:** Demographic of participants in system evaluation (healthy volunteers and CF patients)

	CF volunteers, *n* = 9	Healthy volunteers, *n* = 13
Age (years), mean (SD)	32.8 (9.9)	30.0 (8.1)
Sex (male/female)	6/3	7/6
Weight (kg), mean (SD)	67.3 (15.1)	66.6 (15.7)
Height (cm), mean (SD)	168.9 (10.8)	167.5 (9.9)
BMI (kg/m^2^), mean (SD)	23.4 (3.3)	23.6 (4.5)

**Fig. 5:**
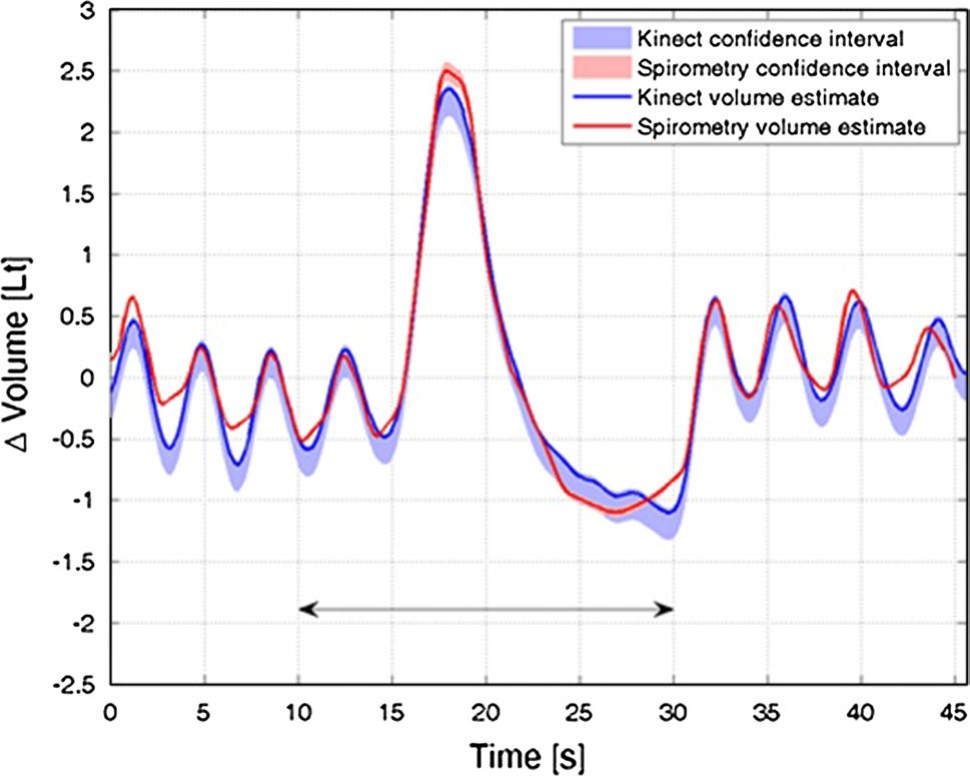
Example dynamic lung volume time series from both Kinect- and spirometry-based estimates

Figure 5 shows a representative example of the Kinect-based system (solid blue line) and spirometry (solid red line) measurements obtained during periods of VC measurement and relaxed breathing in an individual subject. Both data series show estimated 95 % CI with the shaded areas. The Kinect sensor CI was derived from the static mannequin results and is clearly asymmetric or skewed towards lower volumes. The spirometry CI was obtained from the *CareFusion* Microlab Spirometer specifications (www.carefusion.com/medical-products/respiratory/cardio-pulmonary-diagnostics/pulmonary-function-testing/spirometers/microlab.aspx, accessed 12 September 2014). Accuracy was reported to be ±3 % in line with ATS/ERS standards.

There was generally good agreement between Kinect-based measurements and spirometry data. Also shown in Fig. 5 is a black arrow indicating the region around the two VC measurements used to calculate the correlation coefficient, used as a similarity measure. This region was defined in all cases by −8 to +12 s, relative to the peak of the VC time course.

**Fig. 6:**
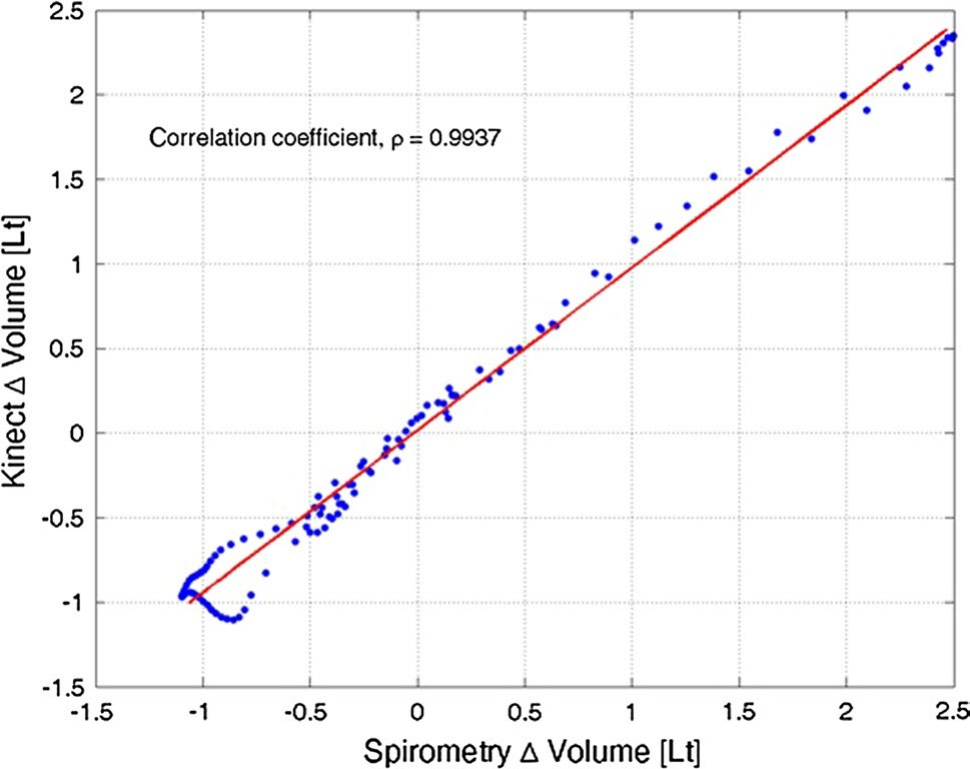
Direct individual time series comparison

Figure 6 shows the aligned point for point comparison of the region of interest (−8 to +12 s window) around the peak in an exemplar relaxed VC measurement, for patient 1, run 2. Indicated in this figure is the estimated correlation coefficient, $\rho = 0.9937$, with upper and lower bounds $\left[ {0.9897, 0.9962} \right],$ showing a significant (*p* < 0.001) correlation between the two time series. Also indicated in this figure by the red line is the total least squares (TLS) regression estimate [15] deemed to be more accurate than standard linear regression, treating the spirometry estimate as the regressor, as both the Kinect and spirometry estimates contain error. The TLS fitting produces an estimate of regression slope and intercept and their respective CI. In the ideal situation, the TLS fit should have+ a slope of 1 and an intercept of 0. In this case, the slope is 0.9601 [0.9580, 0.9621] and the intercept is 0.0167 [0.0147, 0.0187]. The residual standard error from the linear fit is 0.0809 l and can be interpreted as a further similarity measure—the lower the error, the better the fit.

Time series comparison of relaxed VC between Kinect-based and spirometry measurements, for patient 1, run 2.

**Fig. 7:**
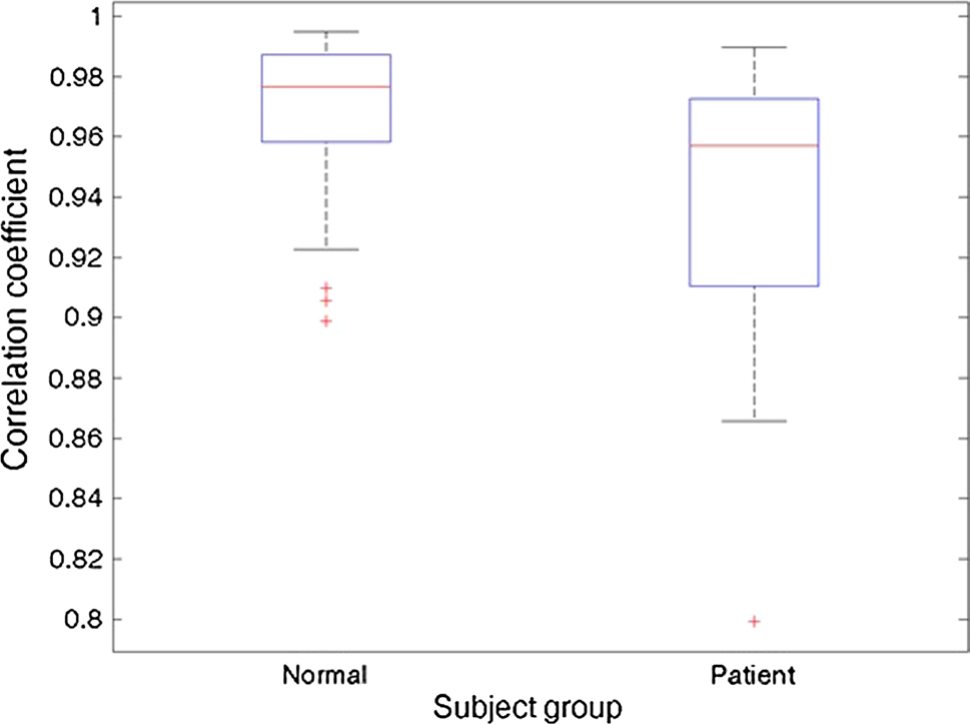
Correlation coefficients between the Kinect-based system and spirometry

Figure 7 shows all of the correlation coefficients obtained between the Kinect-based and spirometry measurements for all subjects and repeated measurements. The figure shows a box-whisker plot for both groups. The central mark indicates the group median values, the edges of the box for the 25th and 75th percentiles and the whiskers extend to the most extreme data points. Identified outliers are shown by the red crosses. All correlation coefficients not deemed outliers were above 0.8656 for the patient group and above 0.9226 for the healthy volunteer group. All correlation coefficients were shown to be significant at a CI of 99 % (*p* values <0.001), where they were computed from a *t* distribution, assuming underlying bivariate normal distribution of the data.

A one-way ANOVA (*F*[1,51] = 7.5783; *p* = 0.0082) indicated that the two groups’ sample means were significantly different. Thus, the CF subjects demonstrated slightly poorer correlation coefficients.

Measurements of dynamic lung volume for all subjects and repeated measurements.

It was observed, but not plotted here for brevity, that there was a small but significant (*p* < 0.001) negative correlation [$\rho = - 0.5243 [ - 0.6960, - 0.2960]$] between the correlation coefficients and the residual error from the TLS fitting procedure. Both the TLS fit and correlation coefficient measures attempt to determine the degree of similarity between the Kinect-based and spirometry measurements. Larger TLS residual error occurs where the two estimates are not well represented by a linear relationship, also indicated by a low correlation coefficient.

Histograms of total least squares regression fitting parameters, intercept and slope, for all dynamic lung volume measurements.

**Fig. 8:**
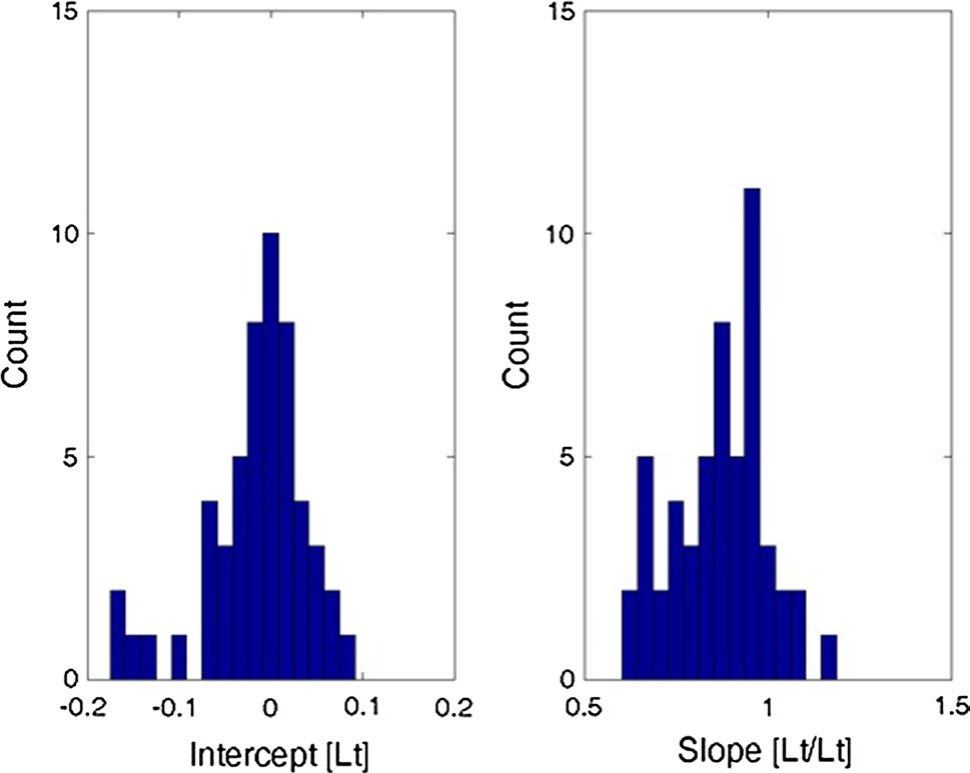
Summary histograms showing fitted intercept and slope estimates from the TLS regression

Figure 8 shows histograms of all the fitted intercept and slope estimates from the TLS regression. Typically, the intercept (left) is grouped around 0. The slopes are skewed to slightly less than unity. As established in the static analysis, the error on the combined Kinect-based system estimates tended towards underestimation rather than overestimation. This is seen in the bias (nonzero mean and median) and the long tail (skewed nature of the extreme value distribution). The TLS slope estimates are less than unity, indicating tendency towards underestimation.

## Discussion

This paper reports the development of a low-cost and portable method for analysing chest wall motion through the use of four Microsoft Kinect devices used in parallel. The system uses a combination of bespoke software and off-the-shelf software, in the form of Geomagic Studio, to evaluate a three-dimensional model of a patient’s torso taken over time and evaluate changes in volume in order to calculate chest wall motion. Static testing and testing on healthy volunteers and cystic fibrosis patients in comparison with spirometry show good correlation (albeit a small underprediction).

The system could prove useful for assessing the effects of respiratory interventions such as medications and physiotherapy techniques. The system also has potential in the pre- and post-operative assessment of thoracic surgical interventions to treat lung conditions including lung cancer, as well as structural abnormalities such as pectus excavatum and paralysed hemidiaphragm. In particular, there is potential for the use of the tool as a tele-assessment device, particularly as there is potential to use the system whilst the patient is seated, or standing.

An advantage of using data from four Kinect sensors is that this system is able to accurately assess chest wall motion even in moving subjects. This is potentially very useful for assessing changes in respiratory physiology that occur on exercise, such as dynamic hyperinflation. This is in contrast to previously described chest wall motion analysis systems that rely on data from one viewpoint [18]. This again allows for more effective use of the system for tele-assessment.

It was previously discussed in this paper that an advantage of using this system is the ability to measure regional motion. Although this was not fully evaluated in this initial testing, the system was designed with this in mind. It is possible, when the point clouds are exported from the bespoke software, to be able to split the point cloud in any way necessary. It is then possible to evaluate the volume of each individual component created, allowing for regional motion analysis.

The system is currently in a prototype stage and shows promising results. However, further development is required to create a fully integrated software which does not involve the use of Geomagic Studio. The hardware itself must also be adapted to be more suitable for the clinical environment (e.g. alleviation of excess wiring, producing single device). With a new version of the Microsoft Kinect being released, and future SDK updates, there is also scope for improvement in these results through the enhancement of the technology itself.

In terms of the accuracy of the system, the probability distribution of the error on the Kinect-based system highlighted a systematic, albeit small, underprediction of static volume. It appears from this testing that the system is accurate when measuring static objects. For the purposes of this testing, it was decided that this underprediction was small enough to conduct dynamic capture successfully. However, in future iterations of the prototype, these data will require further investigation. With further work on the calibration process, and refinement of the algorithms used for smoothing, this error may be reduced.

Full system testing with 13 healthy volunteers and nine CF was conducted to assess dynamic capture capabilities. Estimates of volume change showed a high correlation with that of spirometry in both groups. Through the total least squares regression, it is shown that Kinect-based measurements and spirometry are in good agreement, with a tendency for Kinect-based measurements to underpredict that of spirometry. This is further in agreement with the static analysis.

There were significant differences between the groups, due to airflow obstruction, characteristic of CF lung disease. This may be down to the original design stage of the system in which testing was performed on healthy volunteers. At this point, there may not have been enough focus on the movement of the diaphragm, and this is something that will need to be addressed in future versions.

## Conclusion

In summary, these initial results suggest that the novel Kinect-based system is an accurate and affordable method of rapidly measuring chest wall motion. Some slight underprediction has been shown in the results, but in general, a promising correlation with spirometry in static objects, healthy volunteers and CF patients has been demonstrated, providing grounds for further work.

Therefore, the research team will be developing the system further, particularly through use of the new version of the Kinect and SDK, and evaluation of the system for testing regional motion and tele-assessments. The team will also be evaluating it in comparison with optoelectronic plethysmography. There will also be further work in CF as well as other respiratory conditions.
